# The Eyes

**Published:** 1892-04

**Authors:** 


### THE EYES.

It has long been known to oculists that when one eye, as a consequence of injury, exhibits a severe inflammation of the iris, that from three to six weeks later, and sometimes even after years, the other uninjured eye becomes inflamed and is almost invariably lost. This disease of the second eye was attributed to sympathy, but it is now known that it is due to the activity of another coccus present in all pus, and first described in 1883, by Professor Rosenbach, of Goettingen, who called it the " golden yellow grape coccus," the term "grape" being suggested by the fact that it exists in clusters resembling a bunch of grapes.

Deutschman raised a pure culture of this grape coccus and injected a little into the eye of an animal. Before long the cocci spread from the eye to the ocular nerve, towards the brain, to the point at which it was intersected by the other ocular nerve which it traversed to the sound eye, where it set up an inflammation similar to what had until then been attributed to sympathy.

The lesson of this discovery is, that since it is impossible to destroy the cocci in the eye first diseased, it is better to remove it at once, especially if the sight is wholly or almost wholly destroyed, so as to guard against the possibility of the migration of the cocci to the sound eye. The chain cocci found in inflammation of the lachrymal gland are not less dangerous than the grape cocci. When pus forms in this gland it extends to the eye, doing no injury as long as the superficial sheathing of the cornea is uninjured; but a particle of the finest dust getting into the eye and scratching the surface renders it accessible to the pus from the lachrymal gland which sets up a most dangerous inflammation of the cornea, which may easily result in the loss of the eye.

It is now well known that it is only through the presence of bacteria that matter or pus makes its appearance in the eye.

Twenty years ago, the most successful oculists were quite unable to account for the fact that after what appeared to be quite a successful operation for cataract, matter made its appearance and the eye was lost. Such an event rarely occurs nowadays, Koch's important discovery that all bacteria are destroyed by a current of steam, having led to the invariable practice of treating surgical instruments in this way before use, to destroy any bacteria that may have alighted on them from the air.

If the cocci once effect a lodgment in the eye, their fabulously rapid rate of increase is such that their removal is almost hopeless. Of so much more importance is the discovery of means to guard against their intrusion.

---

### RARE BEEFSTEAK.

For years I have refused to accept as articles of food what hunters call "wild game." It always seemed to me a sin to take the life of innocent creatures. Man's lower nature needs training, or cultivation from its cannibalism. My last lesson was taken a few weeks since, during a trip to our nearest railroad freighting point. Caring little for a morning meal, it has usually been my custom, through advice of so-called professionally advanced or educated minds, to take at most a small, rare beefsteak. Quite early one morning my peacefully slumbering hours were broken in upon by the most agonizing, pitiful bellowing of cattle. Being at a railroad hotel, I comprehended immediately the cause,—cars freighted with poor, helpless, thirsty cattle, packed so densely that there was no turning around or change of tired position, save as they stood upright and scrambled over each other in a wild affrighted manner. I was struck most forcibly with the lower life's great inconsistencies with higher growth.

Stopping on my way to breakfast, I inquired of a man if the cattle bellowed because of thirst. His reply was, "No, the law is now such that they are compelled to water twice a day; they used to go until they sometimes died on the way without water. But they seem to feel or know that they are destined for the slaughter, and so plead to be free." So it seemed to me. I felt sick in sympathy, a disgust with myself and all humanity, at such unmanly, cruel deeds. Going in to breakfast, my usual small demand was brought, as the waiter has soon learned that it was always the same, but when it came, and I looked upon a slice of beef lying in its red juice, which a short time before had been its life blood, a feeling of such utter disgust at self came over me that I said, "Oh, take it away!" and since that morning I find that food which has quivered with the joy of life, and suffered with conscious agonies of death, is not for me, and cannot further aid my growth. I find that fruit, vegetables, milk and eggs do me far better service.—*The Esoteric.*

